# Evaluation of four primer sets for analysis of comammox communities in black soils

**DOI:** 10.3389/fmicb.2022.944373

**Published:** 2022-07-26

**Authors:** Xin Bai, Xiaojing Hu, Junjie Liu, Haidong Gu, Jian Jin, Xiaobing Liu, Guanghua Wang

**Affiliations:** ^1^Key Laboratory of Mollisols Agroecology, Northeast Institute of Geography and Agroecology, Chinese Academy of Sciences, Harbin, China; ^2^University of Chinese Academy of Sciences, Beijing, China

**Keywords:** comammox, primer, Ntsp-amoA 162F/359R, comamoA F/R, CA/B377f/C576r

## Abstract

Comammox, as a newly discovered ammonia oxidizer, urgently needs highly efficient and specific primers to detect its community structure and diversity. In this study, the performance of widely used primer set Ntsp-amoA 162F/359R and newly designed primer sets comamoA F/R, CA377f/C576r, and CB377f/C576r were evaluated, for high-throughput sequencing of comammox *amoA* genes in natural and arable soils sampled from two locations in the black soil region of northeast China. Results showed that, compared with the primer set comamoA F/R, primers Ntsp-amoA 162F/359R had more advantages in detecting comammox operational taxonomic unit (OTU) numbers, diversity, and community structure. The primer sets CA377f/C576r and CB377f/C576r had an advantage in detecting comammox sequences with low relative abundance. In addition, the results of the phylogenetic tree and the relative abundance of dominant OTUs showed that the comammox in the black soils of northeast China was dominated by *Nitrospira* Clade B. Furthermore, our study found that long-term land use reduced the alpha diversity of the comammox community, but lead to the convergent evolution of community structure. The Mantel test and canonical correspondence analysis indicated that soil NO_3_^–^-N content was the most important factor affecting the community structure of comammox. Our study provided experience accumulation for the selection of comammox primers for high-throughput sequencing in the black soil of northeast China.

## Introduction

Nitrification, the oxidation of ammonia to nitrate, is an important part of the global nitrogen cycle (Kowalchuk and Stephen, 2001). Traditional nitrification was thought to be a two-step process that was completed by two types of microorganisms with different functions. They are ammonia-oxidizing microorganisms (AOM), including ammonia-oxidizing bacteria (AOB) and ammonia-oxidizing archaea (AOA), which oxidize ammonia to nitrite; and nitrite-oxidizing bacteria (NOB), which oxidize nitrite to nitrate (Mertens et al., 2009). Comammox, a newly discovered AOM, can synchronously carry out ammonia oxidation, and nitrite oxidation process and oxidize ammonia to nitrate in a single organism (Daims et al., 2015). The discovery of comammox has changed the researchers’ understanding of the nitrification process, and the relative nitrification contribution of different nitrifying microorganisms in the environment needs to be re-evaluated. At present, the research on comammox has become a hot topic in the field of nitrifying microorganisms (Jiang et al., 2019; Li et al., 2019; Li C. et al., 2020).

By metagenomic sequencing analysis, the ammonia monooxygenase gene (*amoA*) of comammox was evolutionarily different from that of AOA and AOB (Daims et al., 2015). Members of comammox belong to *Nitrospira* lineage II, including two branches of Clade A and Clade B (Daims et al., 2015). Clade A and Clade B are further divided into Clades A.1 and A.2 (Xia et al., 2018), and Clades B.1 and B.2 (Lin et al., 2020). At present, the use of molecular biological methods to study comammox community diversity by specific PCR primers has been commonly applied in complex environment samples (Zhao et al., 2019; Takahashi et al., 2020; Wang et al., 2021). The best primers should be able to amplify all members of the comammox and exclude sequences from other organisms. To date, there have been several primer sets designed for PCR amplification of comammox *amoA* gene. For example, the primer sets comA-244F/659R and comB-244F/659R designed by Pjevac et al. (2017), and the primer sets CA377f/C576r and CB377f/C576r newly designed by Jiang et al. (2020) could be used to amplify comammox *Nitrospira* Clade A and Clade B, separately. The specific primer set Ntsp-amoA 162F/359R designed by Fowler et al. (2018), and primer set comamoA F/R designed by Zhao et al. (2019) can amplify both comammox *Nitrospira* Clade A and Clade B. To date, the primer sets Ntsp-amoA 162F/359R and comA/B-244F/659R have been widely used to detect the abundance and community diversity of comammox in various environmental samples (Liu et al., 2019; Roots et al., 2019; Wang J. et al., 2019; Xu Y. et al., 2020; Liu et al., 2021). In addition, there are other specific primers, such as A378f/C616r and comamoA AF/SR, which can only amplify the comammox *Nitrospira* Clade A (Xia et al., 2018; Wang et al., 2018). Although several primer sets were reported, results of a few studies showed that specific amplification products could not be obtained using the primer sets comA/B-244F/659R in some pasture, arable soil, and wetland soil samples (Li et al., 2019; Li S. et al., 2020; Lin et al., 2020). Using primer set CA/B377f/C576r, Jiang et al. (2020) only obtained PCR products of comammox *Nitrospira* Clade A but not comammox *Nitrospira* Clade B from aerobic active sludge estuary sediment, and intertidal soil samples. In another study, Harringer and Alfreider (2021) found that the primer set Ntsp-amoA 162F/359R produced non-specific amplification in activated sludge, rainwater tank, field, and compost soil samples. Therefore, these primers may have some limitations in the detection of comammox communities in different environments. It is still uncertain which primers have higher coverage and specificity for PCR amplification of the comammox *amoA* gene in the soil environment.

The Northeast black soil region is one of the most important commodity grain production bases in China (Liu et al., 2010). The high organic matter content and extensive fertilization in the black soil region suggest that it may contain a rich diversity of AOM. In a previous study, we investigated the abundance and diversity of AOA and AOB in the black soil region of northeast China (Liu et al., 2018), but the comammox community is largely unknown. In this study, we chose the widely used primer set Ntsp-amoA 162F/359R and the newly designed primer sets comamoA F/R, CA377f/C576r, and CB377f/C576r for targeting the comammox *amoA* gene. The diversity and composition of comammox communities in natural and arable soils sampled from two locations in the black soil region of northeast China were analyzed with high-throughput amplicon sequencing. The purposes of this study were (1) to test which comammox primers are more suitable for high-throughput sequencing in the black soils and (2) to investigate whether land use has changed the comammox community structure in northeast China.

## Materials and methods

### Soil samples used in this study

Soil samples were collected from Hongwuyue farm (48°46′7′′N, 125°31′54′′E) and Zhaoguang farm (48°0′35′′N, 126°58′31′′E) in Heilongjiang province in October 2019. At each location, arable soil and nearby natural soil were collected synchronously. The natural soils in the Hongwuyue farm and Zhaoguang farm were named N1 and N2; while the arable soils in the two farms were named F1 and F2, respectively. Each soil (treatment) was collected with three replicates, and each replicate contained mixed soil samples of 0–20 cm depths which were randomly collected from five sites. Archived air-dried soils sieved through 2-mm mesh were used for this study (Liu et al., 2021). Briefly, the air-dried soils were adjusted to 25% mass water content (simulate the soil in natural conditions) and preincubated at 25°C for 7 days to restore microbial activity (Zhang et al., 2019). The preincubated soils were used for DNA extraction and the determination of soil properties. The contents of soil pH, total carbon (TC), total nitrogen (TN), total potassium (TK), total phosphorus (TP), available potassium (AK), available phosphorus (AP), NH_4_^+^-N, and NO_3_^–^-N were determined by the methods described previously (Hu et al., 2017). The detailed sample information and soil properties are shown in Table 1.

**TABLE 1:** Soil properties and geographic information of each soil sample.

Sample name	Soil type	Location	pH	NO_3_^–^-N (mg kg^–1^)	NH_4_^+^-N (mg kg^–1^)	TC (g kg^–1^)	TN (g kg^–1^)	TP (g kg^–1^)	TK (g kg^–1^)	AP (mg kg^–1^)	AK (mg kg^–1^)
N1	Tessellated meadow	Hongwuyue farm	5.05 ± 0.28ab	11.56 ± 1.59c	47.51 ± 4.51b	60.40 ± 3.25b	5.04 ± 0.37b	1.18 ± 0.06c	18.62 ± 0.55a	16.38 ± 3.41b	234.90 ± 19.53a
F1	Farmland (corn)	Hongwuyue farm	4.86 ± 0.07b	28.04 ± 2.35b	31.16 ± 0.06b	40.20 ± 0.56c	3.66 ± 0.10c	1.26 ± 0.04bc	17.85 ± 0.72ab	30.74 ± 3.87a	129.28 ± 6.46b
N2	Tessellated meadow	Zhaoguang farm	5.30 ± 0.18a	2.07 ± 0.20d	100.00 ± 25.84a	83.37 ± 13.14a	6.76 ± 1.28a	1.80 ± 0.11a	15.60 ± 0.84c	17.52 ± 2.22b	130.10 ± 10.50b
F2	Farmland (corn)	Zhaoguang farm	4.74 ± 0.07b	34.49 ± 4.21a	28.84 ± 1.70b	54.08 ± 1.90bc	4.92 ± 0.20bc	1.33 ± 0.03b	16.66 ± 0.70bc	19.98 ± 1.80b	114.58 ± 3.26b

TC, total carbon; TN, total nitrogen; TP, total phosphorus; TK, total potassium; AP, available phosphorus; AK, available potassium.

All values were given as mean ± SD; different letters in each column are significantly different at p < 0.05 by analysis of one-way ANOVA.

### Soil DNA extraction and PCR amplification of comammox *amoA* genes

Soil total DNA was extracted from 0.5 g of fresh soil using a FastDNA^®^ Spin Kit for soil (MP Biomedicals, United States) according to the manufacturer’s instructions. The quantity and concentration of extracted DNA were measured by a NanoDrop 2000 spectrophotometer (NanoDrop Technologies, United States), and then the DNA was stored in a −20°C freezer.

Widely used primers Ntsp-amoA 162F/359R (Fowler et al., 2018) and recently designed primers comamoA F/R (Zhao et al., 2019), CA377f/C576r, and CB377f/C576r (Jiang et al., 2020) were selected in this study. Primer sets Ntsp-amoA 162F/359R and comamoA F/R were designed to simultaneously amplify both comammox *Nitrospira* Clade A and Clade B, with PCR product lengths of 198 and 436 bp, respectively (Fowler et al., 2018; Zhao et al., 2019). CA377f/C576r and CB377f/C576r were designed to separately amplify comammox *Nitrospira* Clade A and Clade B, respectively, and both primer sets generated a 200 bp length fragment (Jiang et al., 2020). The specific primer-binding sites with comammox *amoA* genes for different primer sets are shown in Supplementary Figure 1. The targeted fragment of primer set comamoA F/R includes primer sets Ntsp-amoA 162F/359R and CA/B377f/C576r, but the fragment generated between Ntsp-amoA 162F/359R and CA/B377f/C576r has no overlapping region.

Amplification of comammox *amoA* genes for high-throughput sequencing was performed on a GeneAmp^®^ PCR System 9700 (Applied Biosystems, United States). The primers used to amplify each sample were connected with a unique 8-bp barcode sequence at the 5′ end. The PCR was performed in triplicate with a volume of 50 μl: 25 μl of 2× EasyTaq PCR SuperMix (TransGen Biotech, Beijing, China), 2 μl of each primer (5 μM), 1 μl of template DNA, and 20 μl of ddH_2_O. The primer sequences and amplification conditions are shown in Supplementary Table 1.

### High-throughput sequencing and analysis

Illumina MiSeq library preparation and sequencing were performed at Majorbio Bio-Pharm Technology (Shanghai, China). Raw fastq files were de-multiplexed and quality filtered using QIIME (version 1.9.1^1^) (Caporaso et al., 2010). The shorter sequences were removed and then all the remaining sequences were considered in the subsequent analyses. UPARSE embedded in QIIME was used to cluster valid sequences into operational taxonomic units (OTUs) with 95% sequence identity (Wang et al., 2020). The representative sequence of each OTU was determined by blasting against the NCBI-nr database, and the sequences with blast hits of *amoA* genes or *pmoA* genes (potential comammox *amoA* gene but misannotated as *pmoA* gene before the year 2015) were kept and exclusively determined as comammox *amoA* genes in the following phylogenetic analysis (Supplementary Tables 2–5; Lin et al., 2020). According to the blast analysis, the percentage of correct *amoA* reads reached 92% (Ntsp-amoA 162F/359R), 67% (comamoA F/R), 91% (CA377f/C576r), and 83% (CB377f/C576r) for each primer set. For alpha and beta diversity analyses, OTU tables were rarefied as per minimum sequencing depth. Amplicon data are deposited into the NCBI Sequence Read Archive (SRA) with accession number PRJNA786474.

### Phylogenetic analysis

The representative sequence of each OTU was aligned with the comammox *amoA* gene sequences using MEGA 11 (Tamura et al., 2021). For each OTU, a representative sequence was chosen and aligned against the NCBI database. A taxonomy information was reconfirmed through an amino acid-based phylogenetic tree constructed with OTU representative sequences and comammox reference sequences using the neighbor-joining method with 1,000 bootstrap iterations. Nucleic acid-based phylogenetic trees were constructed using the same method with OTU representative sequences between two different primer sets.

### Statistics analysis

The measured soil property data and alpha diversity indexes were subjected to analysis of variance with one-way ANOVA, followed by Fisher’s least significant differences (LSD) test (α = 0.05) to determine the differences among the treatments (SPSS 25 for Windows, IBM Corp., Armonk, NY, United States). Principal coordinate analysis (PCoA) based on Bray–Curtis distance was conducted to investigate the beta diversity of comammox communities in different soil samples. The Mantel test and canonical correspondence analysis (CCA) were performed to explore the influence of environmental factors on the comammox community. PCoA, mantel test, and CCA were all performed using the “vegan” package in the R environment (version 4.1.2) (R Development Core Team, 2016).

## Results

### Soil properties

In each sampling site, most of the soil properties between arable and natural soils were significantly different (Table 1). The contents of soil pH, TC, TN, AK, and NH_4_^+^-N in farmland soils were lower, while soil AP and NO_3_^–^-N content were higher than those properties in natural soils.

### Diversity of comammox *amoA* genes

Totally, 48 PCR products (4 treatments × 3 replicates × 4 primer sets) were successfully obtained in this study. After MiSeq sequencing and quality control, 151,740 and 43,236 comammox *amoA* sequences were generated with primer sets Ntsp-amoA 162F/359R and comamoA F/R, respectively; 50,220 sequences of comammox *Nitrospira* Clade A and 29,208 sequences of comammox *Nitrospira* Clade B were generated with primer sets CA377f/C576r and CB377f/576r, respectively. Rarefaction curves showed that the sequencing depth was sufficient for diversity analysis of comammox *amoA* genes (Supplementary Figure 2). In total, 75 and 56 OTUs were identified with the primer sets Ntsp-amoA 162F/359R and comamoA F/R, respectively; while 52 and 59 OTUs were detected with the primer sets CA377f/C576r and CB377f/576r, respectively.

Using primer set Ntsp-amoA 162F/359R, the alpha diversity indexes, including Shannon diversity, Chao1, and OTU number in N1 and N2, were significantly higher than those in F1 and F2 (Table 2). The change of alpha diversity indexes between samples with primers comamoA F/R was similar with primers Ntsp-amoA 162F/359R, but there was no significant difference in Shannon index among different soils. Using primer set CA377f/C576r, Shannon index of comammox *Nitrospira* Clade A was the highest in N2, followed by N1, and low in F1 and F2; Chao1 index and OTU number in N2 were significantly higher than those in other samples. For primer set CB377f/C576r, the comammox *Nitrospira* Clade B diversity, richness index, and OTU number in N1 and N2 were higher than those in F1 and F2.

**TABLE 2:** Alpha diversity indexes with different primer sets in different soil samples.

Primer name	Sample name	OTUs	Chao1	Shannon
Ntsp-amoA 162F/359R	N1	44 ± 2a	45 ± 1a	2.11 ± 0.19a
	F1	27 ± 2b	29.0 ± 1b	1.30 ± 0.49b
	N2	44 ± 2a	48 ± 4a	2.07 ± 0.11a
	F2	28 ± 5b	29 ± 6b	1.29 ± 0.25b
comamoA F/R	N1	27 ± 4a	29 ± 2a	1.65 ± 0.24a
	F1	16 ± 2b	17 ± 2b	1.32 ± 0.54a
	N2	29 ± 7a	32 ± 9a	1.83 ± 0.12a
	F2	13 ± 2b	13 ± 2b	1.42 ± 0.01a
CA377f/C576r	N1	16 ± 4b	16 ± 4b	1.43 ± 0.11b
	F1	13 ± 1b	13 ± 1b	0.51 ± 0.07c
	N2	32 ± 3a	34 ± 2a	2.28 ± 0.14a
	F2	14 ± 3b	14 ± 3b	0.58 ± 0.33c
CB377f/C576r	N1	26 ± 3b	28 ± 1b	2.11 ± 0.12a
	F1	13 ± 1c	14 ± 3c	1.36 ± 0.08b
	N2	33 ± 3a	40 ± 6a	2.39 ± 0.15a
	F2	12 ± 3c	14 ± 5c	1.02 ± 0.27c

N1 and F1 are natural soils and arable soils in Hongwuyue farm, respectively; N2 and F2 are natural soils and arable soils in Zhaoguang farm, respectively.

All values were given as mean ± SD; different letters with each primer set are significantly different at p < 0.05 by analysis of one-way ANOVA.

### Comammox community composition

**FIGURE 1:**
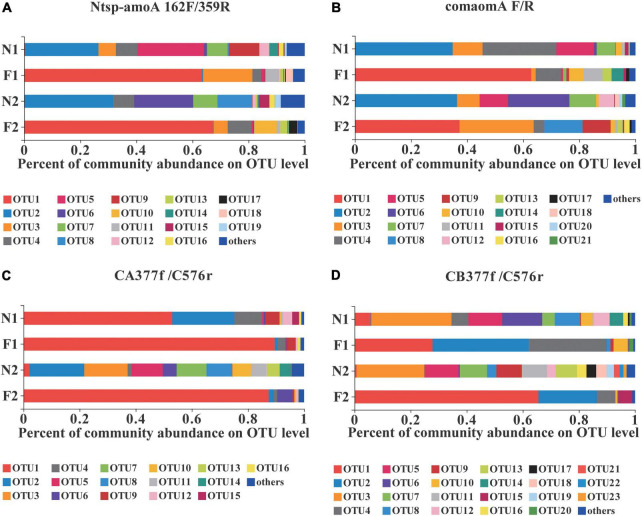
Relative abundance of dominant comammox OTUs on average in each sample by primer sets **(A)** Ntsp-amoA 162F/359R, **(B)** comamoA F/R, **(C)** CA377f/C576r, and **(D)** CB377f/C576r. OTUs with a relative abundance less than 1% in all samples were classified as others. N1 and F1 are natural and arable soils in Hongwuyue farm, respectively; N2 and F2 are natural and arable soils in Zhaoguang farm, respectively. Each sample is based on the average of three replicates.

**FIGURE 2:**
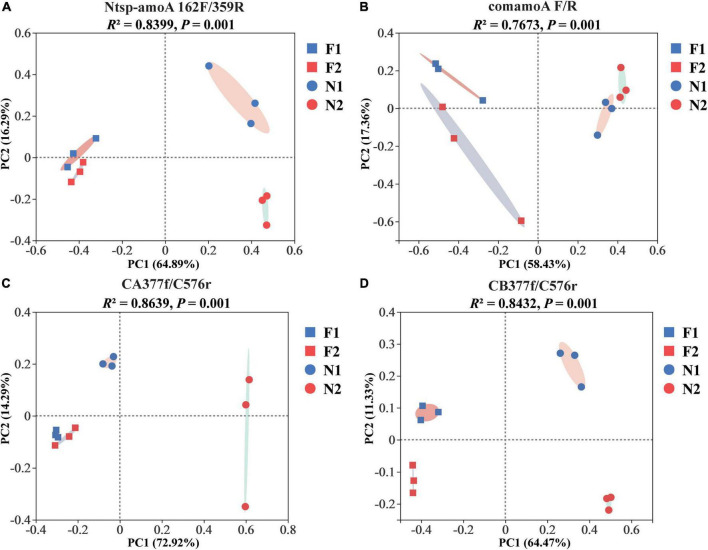
Principal coordinate analysis (PCoA) of comammox community dissimilarities among different samples by primer sets **(A)** Ntsp-amoA 162F/359R, **(B)** comamoA F/R, **(C)** CA377f/C576r, and **(D)** CB377f/C576r using Bray–Curtis distance. N1 and F1 are natural and arable soils in Hongwuyue farm, respectively; N2 and F2 are natural and arable soils in Zhaoguang farm, respectively.

At the OTU level, comammox community composition showed the difference in dominant OTUs between samples (Figure 1). The PCoA plots revealed that the total comammox communities were well separated along the first two principal coordinate axes according to the land use examined by primer sets Ntsp-amoA 162F/359R (Adonis: *R*^2^ = 0.8399, *p* = 0.001) and comamoA F/R (Adonis: *R*^2^ = 0.7673, *p* = 0.001) (Figures 2A,B). Comammox *Nitrospira* Clade A and Clade B communities were also separated according to the land use by primer sets CA377f/C576r (Adonis: *R*^2^ = 0.8639, *p* = 0.001) and CB377f/C576r (Adonis: *R*^2^ = 0.8432, *p* = 0.001), respectively (Figures 2C,D). In addition, the similarity of communities between the two arable soils was higher than that of the two natural soils, except for the results of using primers comamoA F/R.

**FIGURE 3:**
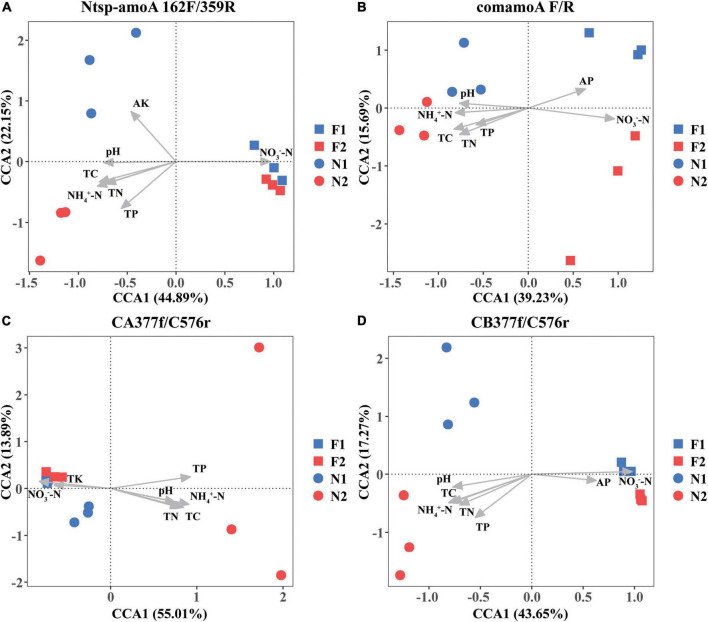
Canonical correspondence analysis (CCA) of changes in comammox communities with environmental variables among different samples by primer sets **(A)** Ntsp-amoA 162F/359R, **(B)** comamoA F/R, **(C)** CA377f/C576r, and **(D)** CB377f/C576r. N1 and F1 are natural and arable soils in Hongwuyue farm, respectively; N2 and F2 are natural and arable soils in Zhaoguang farm, respectively.

The Mantel test analysis for using four primer sets showed that soil pH, NH_4_^+^-N, NO_3_^–^-N, TC, TN, and TP were all significantly correlated with comammox community structures. Specifically, soil NH_4_^+^-N and NO_3_^–^-N were the two dominating factors when using primer sets Ntsp-amoA 162F/359R and CB377f/C576r, and soil NO_3_^–^-N and TC were the two major factors shifting comammox community structures by using primer set comamoA F/R, while soil TP and NH_4_^+^-N were two major factors shifting comammox community structures using primer set CA377f/C576r (Table 3). In addition, the plots of CCA with different primer sets were consistent with the results of the mantel test (Figure 3).

**TABLE 3:** Mantel test results for the correlation between comammox community composition and environmental variables with different primer sets.

Variable	Ntsp-amoA 162F/359R		comamoA F/R		CA377f/C576r		CB377f/C576r	
	*r*	*p*	*R*	*p*	*r*	*p*	*r*	*p*
pH	0.4364	0.005	0.2885	0.020	0.5368	0.007	0.4309	0.005
NH_4_^+^-N	0.5386	0.001	0.3543	0.001	0.8426	0.001	0.5320	0.001
NO_3_^–^-N	0.8214	0.001	0.7472	0.001	0.6761	0.001	0.8705	0.001
TC	0.4394	0.001	0.3996	0.001	0. 6740	0.001	0.4631	0.001
TN	0.3231	0.006	0.3110	0.007	0.5781	0.005	0.3520	0.005
TP	0.4575	0.001	0.2093	0.037	0.8753	0.001	0.4336	0.002
AP	0.1524	0.130	0.2319	0.026	−0.0721	0.596	0.2070	0.046
TK	0.2256	0.055	0.0168	0.344	0.4341	0.009	0.1959	0.063
AK	0.3114	0.034	0.0578	0.266	−0.018	0.452	0.2028	0.052

TC, total carbon; TN, total nitrogen; TP, total phosphorus; TK, total potassium; AP, available phosphorus; AK, available potassium.

### Phylogenetic analysis

**FIGURE 4:**
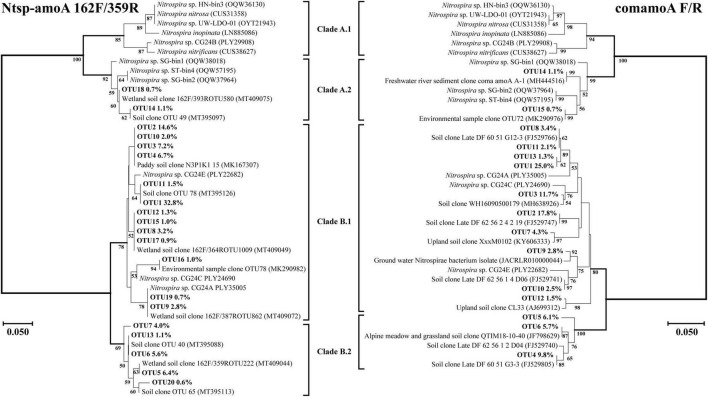
Phylogenetic trees of comammox *amoA* gene sequences at amino acid level by primer sets Ntsp-amoA 162F/359R and comamoA F/R. Percentages following the OTUs indicate the proportion of each OTU to the total comammox *amoA* gene sequences. Only OTUs with more than 0.5% of the obtained comammox *amoA* gene sequences are shown in the phylogenetic tree. Phylogenetic analysis was performed using the neighbor-joining method with 1,000 bootstraps. The scale bar represents 5% amino acid sequence divergence, and the bootstrap values (>50%) are shown at branch points.

**FIGURE 5:**
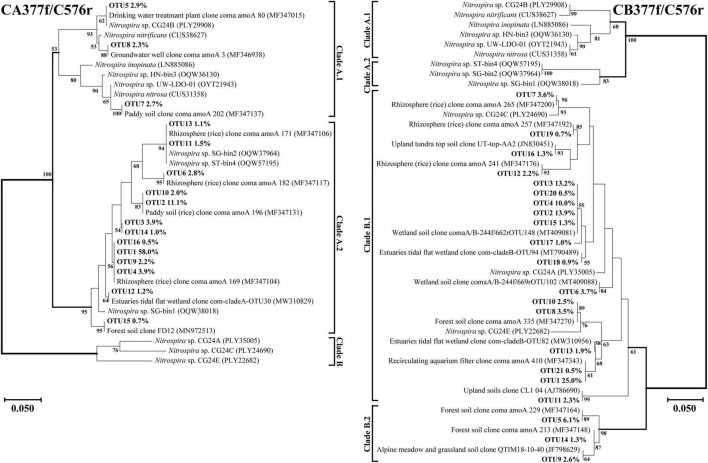
Phylogenetic trees of comammox *amoA* gene sequences at amino acid level by primer sets CA377f/C576r and CB377f/C576r. Percentages following the OTUs indicate the proportion of each OTU to the total comammox *amoA* gene sequences. Only OTUs with more than 0.5% of the obtained comammox *amoA* gene sequences are shown in the phylogenetic tree. Phylogenetic analysis was performed using the neighbor-joining method with 1,000 bootstraps. The scale bar represents 5% amino acid sequence divergence, and the bootstrap values (>50%) are shown at branch points.

Amino acid-based phylogenetic trees of dominant comammox *amoA* OTUs (relative abundance of each out >0.5%) from all soil samples were constructed using the neighbor-joining method (Figures 4, 5). The closest relative of all dominant OTUs in Figures 4, 5 is presented in Supplementary Tables 2–5, and the results indicated that the dominant OTUs of this study had more than 97% identity with the sequences deposited in the NCBI database (Supplementary Tables 2–5).

Both Clade A and Clade B of comammox *Nitrospira* were detected with the primer sets of Ntsp-amoA 162F/359R and comamoA F/R (Figure 4). Clade A and Clade B were further divided into two clades, Clade A.1 and Clade A.2, Clade B.1 and Clade B.2, respectively. Using primer set Ntsp-amoA 162F/359R, 20 OTUs were detected as the dominant members, of which 13 and 5 OTUs were classified as Clade B.1 and Clade B.2, accounting for 75.7 and 17.7% of the total *amoA* gene sequence numbers, respectively; two OTUs were classified as Clade A.2, accounting for 1.8% of the total *amoA* gene sequence numbers. Using the primers comamoA F/R, 15 OTUs were identified as the dominant members. Among them, 10 and 3 OTUs were classified as Clade B.1 and Clade B.2, which accounted for 72.3 and 21.6% of the total sequence numbers, respectively. Two OTUs were classified as Clade A.2, accounting for 1.8% of the sequence numbers.

Using primer set CA377f/C576r, 16 OTUs were determined as the dominant members, which were all classified into comammox *Nitrospira* Clade A (Figure 5). Among them, 3 OTUs were classified as Clade A.1, accounting for 7.9% of the total *amoA* gene sequence numbers, and 13 OTUs were classified as Clade A.2, accounting for 90.0% of the total *amoA* gene sequence numbers. Differently, using the primer set CB377f/C576r, all OTUs were grouped into comammox *Nitrospira* Clade B, and 21 OTUs were identified as the dominant members. Among them, 18 and 3 OTUs were classified as Clade B.1 and Clade B.2, accounting for 87.9% and 10.0% of the total *amoA* gene sequence numbers, respectively.

### Comparison of specificity among different primer sets

Blast alignment was performed for each OTU, and then phylogenetic classification was performed for each sample sequenced by the four primer sets (Supplementary Table 6). Results showed that the relative abundances of comammox *Nitrospira* Clade A and Clade B detected by primer sets Ntsp-amoA 162F/359R and comamoA F/R were similar, and comammox *Nitrospira* Clade B was dominant in all samples. Moreover, at the nucleic acid level, phylogenetic trees constructed by dominant OTUs generated between primers comamoA F/R and Ntsp-amoA 162F/359R and between primers comamoA F/R and CA/B377f/C576r are shown in Supplementary Figures 3, 4. Most of the dominant OTUs detected by the primer set comamoA F/R were the same as those detected by Ntsp-amoA 162F/359R and CA/B377f/C576r in genetic distance.

## Discussion

The selection of primers with wide coverage, high specificity, and high amplification efficiency is the critical step to study the ecological and functional characteristics of comammox communities. To provide a reliable basis for primer selection as far as possible, we compared the performance of the commonly used primer set Ntsp-amoA 162F/359R and the newly designed primer sets comamoA F/R and CA/B377f/C576r for analyzing comammox community in two natural and two arable black soils. Although all primer sets successfully amplified comammox *amoA* genes for high-throughput sequencing, there were still some differences in terms of alpha diversity, beta diversity, non-specific amplification, and correlations with environmental variables among different primers.

### Comparison of alpha diversity of comammox using different primer sets

The primer set Ntsp-amoA 162F/359R was originally designed to detect comammox in drinking water treatment plants (Fowler et al., 2018). Subsequently, this primer set was widely applied to the determination of comammox abundance and community structure in nitrification reactors (Roots et al., 2019), agricultural soils (Wang J. et al., 2019; Li S. et al., 2020; Wang et al., 2020), lake sediments (Xu Y. et al., 2020), and lakes (Harringer and Alfreider, 2021). The primer set comamoA F/R designed by Zhao et al. (2019) successfully detected comammox in agricultural soils, river sediments, drinking waters, intertidal zones, and activated sludges and achieved a higher coverage rate than the primer set Ntsp-amoA 162F/359R in databases. Besides, the 436 bp amplification length generated with primers comamoA F/R was considered to provide a perfect size for paired-end high-throughput sequencing at the Illumina Miseq platform. However, in this study, compared with comamoA F/R, more OTU numbers were observed with Ntsp-amoA 162F/359R in all soil samples (Table 2). Under the similar variation trend of alpha diversity index among samples, no significant difference in the Shannon index among soil samples was detected by comamoA F/R. These findings indicated that comamoA F/R might have a low sensitivity in detecting the comammox OTUs with low abundances, leading to few differences in comammox diversity among different samples.

The primer sets CA/B377f/C576r could amplify comammox *Nitrospira* Clade A and comammox *Nitrospira* Clade B, respectively. Those primer sets can achieve a higher coverage than the primer set Ntsp-amoA 162F/359R and primer sets comA/B-244F/659R that also can respectively amplify comammox *Nitrospira* Clade A and comammox *Nitrospira* Clade B (Pjevac et al., 2017; Jiang et al., 2020). In this study, although the OTU numbers generated from primer sets CA377f/C576r and CB377f/C576r were fewer than those from primers Ntsp-amoA 162F/359R, the total OTU numbers generated from primers CA/B377f/576r (Clade A + Clade B) were similar to or higher than primer set Ntsp-amoA 162F/359R in all treatments (Table 2).

In addition, although the alpha diversities of comammox communities were detected differently by using four primer sets, it is worthy to note that the alpha diversity indexes in natural soils were higher than that in adjacent arable soils. This finding suggested that long-term land use may reduce the alpha diversity of comammox communities. This phenomenon needs to be confirmed with more evidence in future study.

### Comparison of beta diversity of comammox using different primer sets

Although four primer sets successfully generated the *amoA* genes of comammox, the soil comammox community structures varied with primer sets. A similar change trend of community structures among soils was observed with the primer sets Ntsp-amoA 162F/359R and CA/B377f/C576r, which showed that the two arable soils were relatively close, which were far away from the two natural soils (Figures 2A,C,D). In contrast, primer set comamoA F/R detected the two natural soils closely grouped, but the two arable soils were far separated (Figure 2B). These findings indicated that the primer sets Ntsp-amoA 162F/359R and CA/B377f/C576r may be more accurate than the primer set comamoA F/R in analyzing soil comammox community structure. In addition, the PCoA results of this study also suggested that long-term land use may lead to the evolution of comammox toward a similar community structure.

Soil pH and ammonium concentration are believed to be the important factors impacting the diversity and activity of canonical ammonia oxidizers in terrestrial environments (Gubry-Rangin et al., 2011; Hu et al., 2014; Prosser and Nicol, 2012). Recently, a study also found that soil pH and NH_4_^+^-N were two key factors in determining the community structure of comammox in Mollisol under long-term fertilization regimes (Sun et al., 2021). However, as a newly discovered nitrifier, the response of comammox community structure to the environmental factors is not widely recognized. In this study, based on primers Ntsp-amoA 162F/359R, comamoA F/R, and CB377f/C576r, we found soil NO_3_^–^-N was the strongest environmental factor affecting the community structure of comammox (Figure 3 and Table 3). Therefore, the responses of comammox community to environmental factors may not be always consistent with those of canonical ammonia oxidizers. Wang et al. (2020) found NO_3_^–^-N had a significant influence on the distribution of comammox in the mudflat and reclaimed agricultural soils, while pH and NH_4_^+^-N exhibited no impact on the comammox community. It is noteworthy that TP and NH_4_^+^-N, rather than NO_3_^–^-N, were the most important environmental factors affecting the community structure of comammox *Nitrospira* Clade A detected by the primer set CA377f/C576r. A previous study found that ammonia transporters of comammox *Nitrospira* Clade A and Clade B were different in genomes (Palomo et al., 2018). Therefore, the community structure of comammox *Nitrospira* Clade A and Clade B may have different responses to environmental variables.

### Comparison of phylogenetic tree using different primer sets

More OTU numbers were detected with primer set Ntsp-amoA 162F/359R than comamoA F/R. After translating the nucleic acid sequence into an amino acid sequence, 20 dominant OTUs detected with Ntsp-amoA 162F/359R were translated into 11 different amino acid sequences in genetic distance; while 15 dominant OTUs detected with comamoA F/R were translated into 13 different amino acid sequences (Figure 4). This result may be attributed to the longer amplification length generated with primer set comamoA F/R (Zhao et al., 2019). However, the topological phylogenetic trees generated between primer sets Ntsp-amoA 162F/359R and comamoA F/R were consistent and comparable. That is, both the primer sets detected two OTUs that accounted for 1.8% of the total sequences belonged to Clade A.2, no OTU belonged to Clade A.1, and the remaining OTUs all belonged to Clade B (Figure 4).

Primer sets CA377f/C576r and CB377f/C576r only detected Clade A and Clade B, respectively, reflecting their high specificity (Figure 5). The primer set CB377f/C576r detected that most of the sequences belonged to Clade B.2, which was consistent with primer sets Ntsp-amoA 162F/359R and comamoA F/R. These findings indicated that the comammox community in the black soils of northeast China was dominated by Clade B. This result was consistent with the previous studies that the abundance of Clade A.2 and Clade B was enriched in agricultural soils and sediments, while Clade A.1 was more commonly found in freshwater, groundwater, and engineered systems (Xu S. et al., 2020; Wang Z. et al., 2019; Wang et al., 2020).

In terms of specific primers, we found that primer sets Ntsp-amoA 162F/359R and CB377f/C576r detected all the correct *amoA* gene sequences belonging to comammox, but primer sets comamoA F/R and CA377f/C576r detected only a few numbers of AOB *amoA* gene sequences in some samples (Supplementary Table 6). As described in a previous study, a few AOB sequences were also detected through high-throughput sequencing by the primer set CA377f/C576r in an aerobic active sludge sample (Jiang et al., 2020). All considered, we recommend choosing appropriate primers for high-throughput sequencing according to the type of environmental samples and research purposes.

## Conclusion

All four primer sets examined in this study could be used for high-throughput sequencing of comammox in the black soils. Compared with the longer PCR product generated with the primer set comamoA F/R, the primer set Ntsp-amoA 162F/359R detected more OTUs and was more advantageous in detecting comammox diversity and community structure. The primer set CA/B377f/C576r could perform high-throughput sequencing of comammox *Nitrospira* Clade A and Clade B, and had an advantage in detecting comammox *Nitrospira* Clade A with low abundance. Through high-throughput sequencing with the above primer sets, we found that the comammox community in two black soil locations of northeast China was dominated by Clade B. Additionally, land use significantly changed the community structure of both comammox *Nitrospira* Clade A and Clade B, and soil NO_3_^–^-N content was the strongest environmental factor affecting the community structure of comammox.

## Data availability statement

The datasets presented in this study can be found in online repositories. The names of the repository/repositories and accession number(s) can be found below: https://www.ncbi.nlm.nih.gov/, PRJNA786474.

## Author contributions

GW and XB contributed to conception of the study. GW, JJ, and XL designed the experiments. XB and JL performed the experiments. HG and XH contributed to interpretation of the results. XB, GW and XH wrote the manuscript. All authors contributed to the article and approved the submitted version.

## Conflict of interest

The authors declare that the research was conducted in the absence of any commercial or financial relationships that could be construed as a potential conflict of interest.

## Publisher’s note

All claims expressed in this article are solely those of the authors and do not necessarily represent those of their affiliated organizations, or those of the publisher, the editors and the reviewers. Any product that may be evaluated in this article, or claim that may be made by its manufacturer, is not guaranteed or endorsed by the publisher.

## Footnotes

1
http://qiime.org/
