# Supplementary material for: Evaluation of four primer sets for analysis of comammox communities in black soils
Source: Front Microbiol. 2022 Jul 26;13:944373. doi: 10.3389/fmicb.2022.944373 (PMC9362984; doi:10.3389/fmicb.2022.944373)
Supplement: Supplementary file 1 [file Data_Sheet_1.DOCX]

***Supplementary Figures***

**
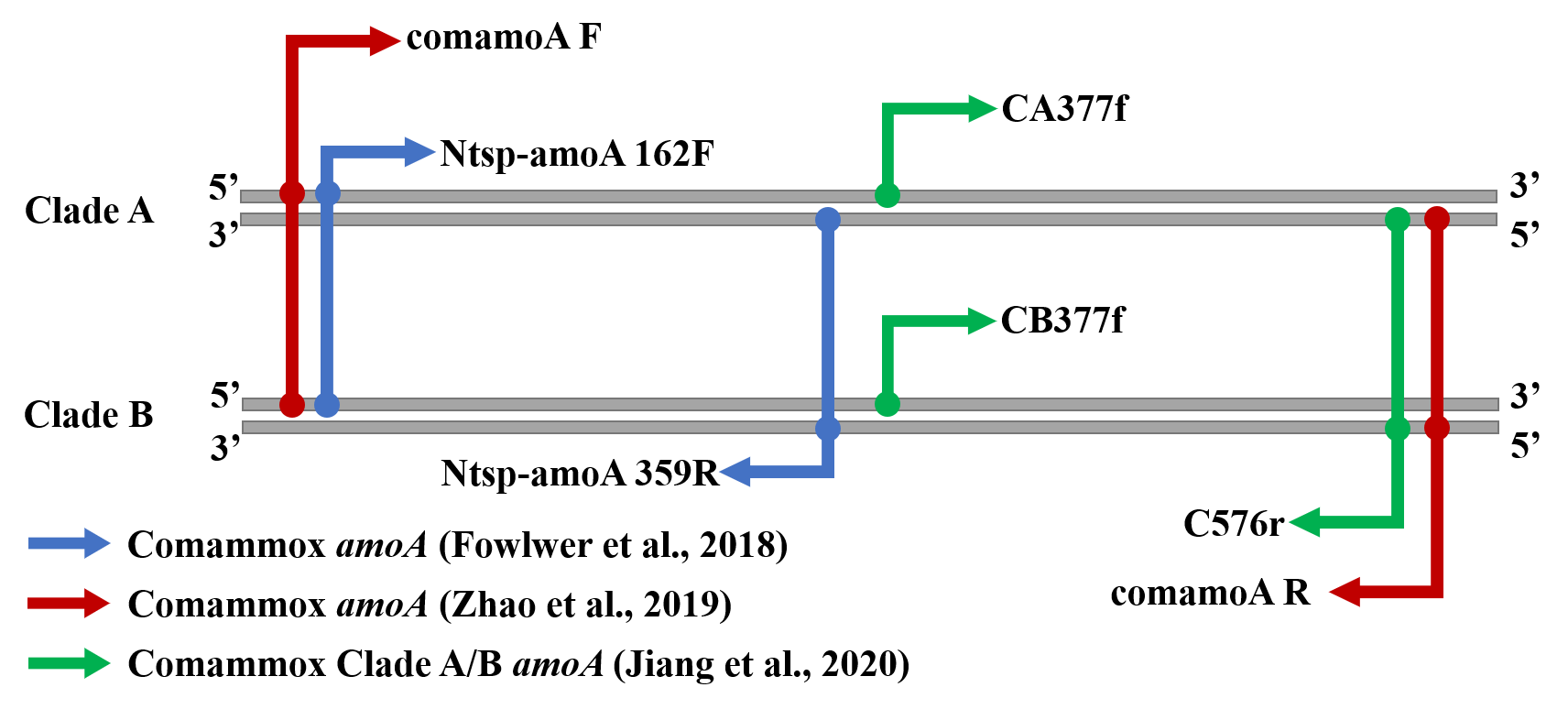
**

**Figure S1** Visual representation of primer-binding sites with comammox Clade A *amoA* genes and comammox Clade B *amoA* genes.


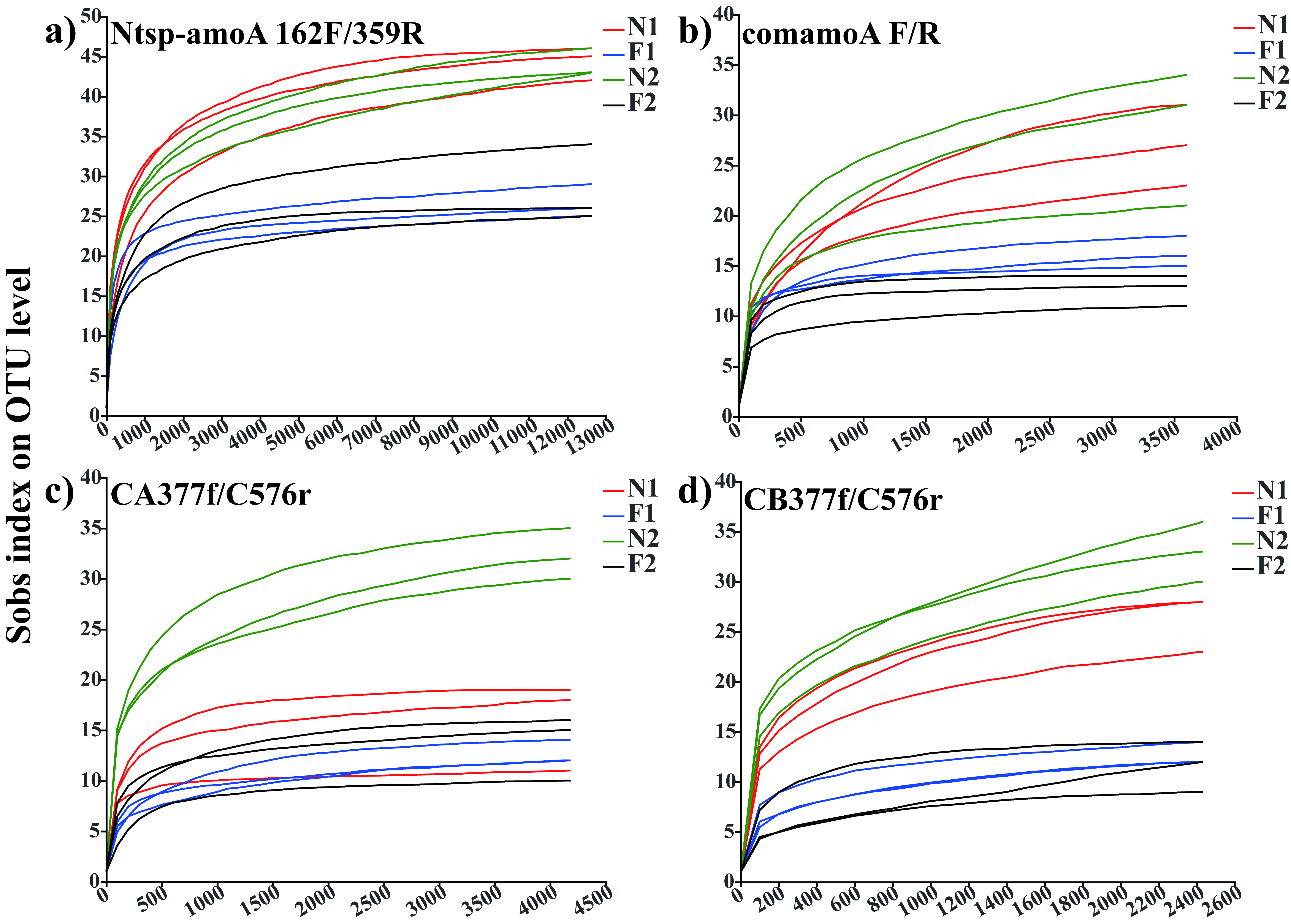


**Figure S2** Rarefaction curves of sobs index on OTU level with primer sets a) Ntsp-amoA 162F/359R, b) comamoA F/R, c) CA377f/C576r and d) CB377f/C576r for high-throughput sequencing. N1 and F1 are natural soils and arable soils in Hongwuyue farm, respectively; N2 and F2 are natural soils and arable soils in Zhaoguang farm, respectively. Each treatment has three replicates.


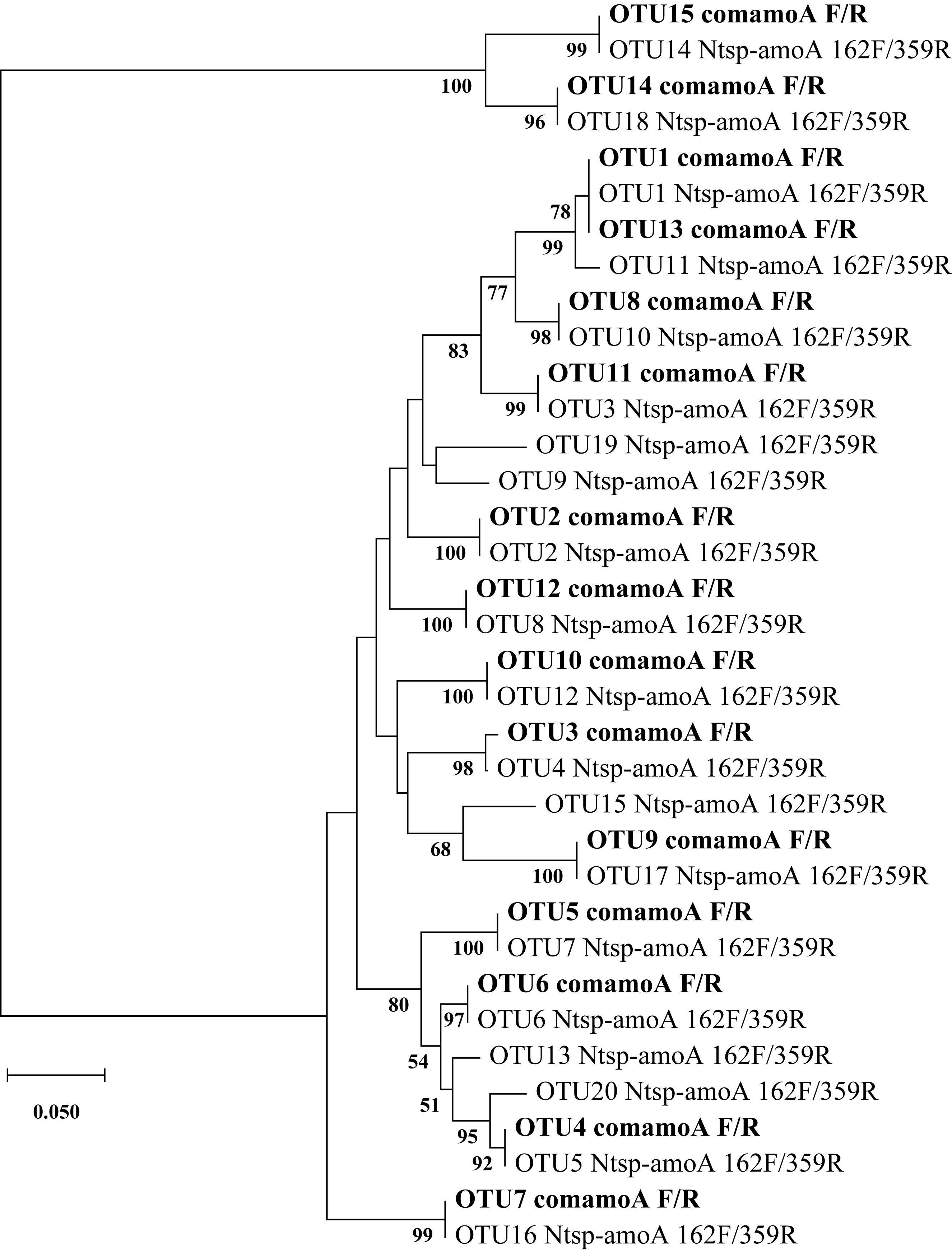


**Figure S3** Phylogenetic tree of comammox *amoA* gene sequences at the nucleotide level detected by primer sets comamoA F/R and Ntsp-amoA 162F/359R. Only OTUs with more than 0.5% of the obtained comammox *amoA* gene sequences are shown in the phylogenetic tree. Phylogenetic analysis was performed using the neighbor-joining method with 1000 bootstraps. The scale bar represents 5% nucleic acid sequence divergence, and the bootstrap values (>50%) are shown at branch points.


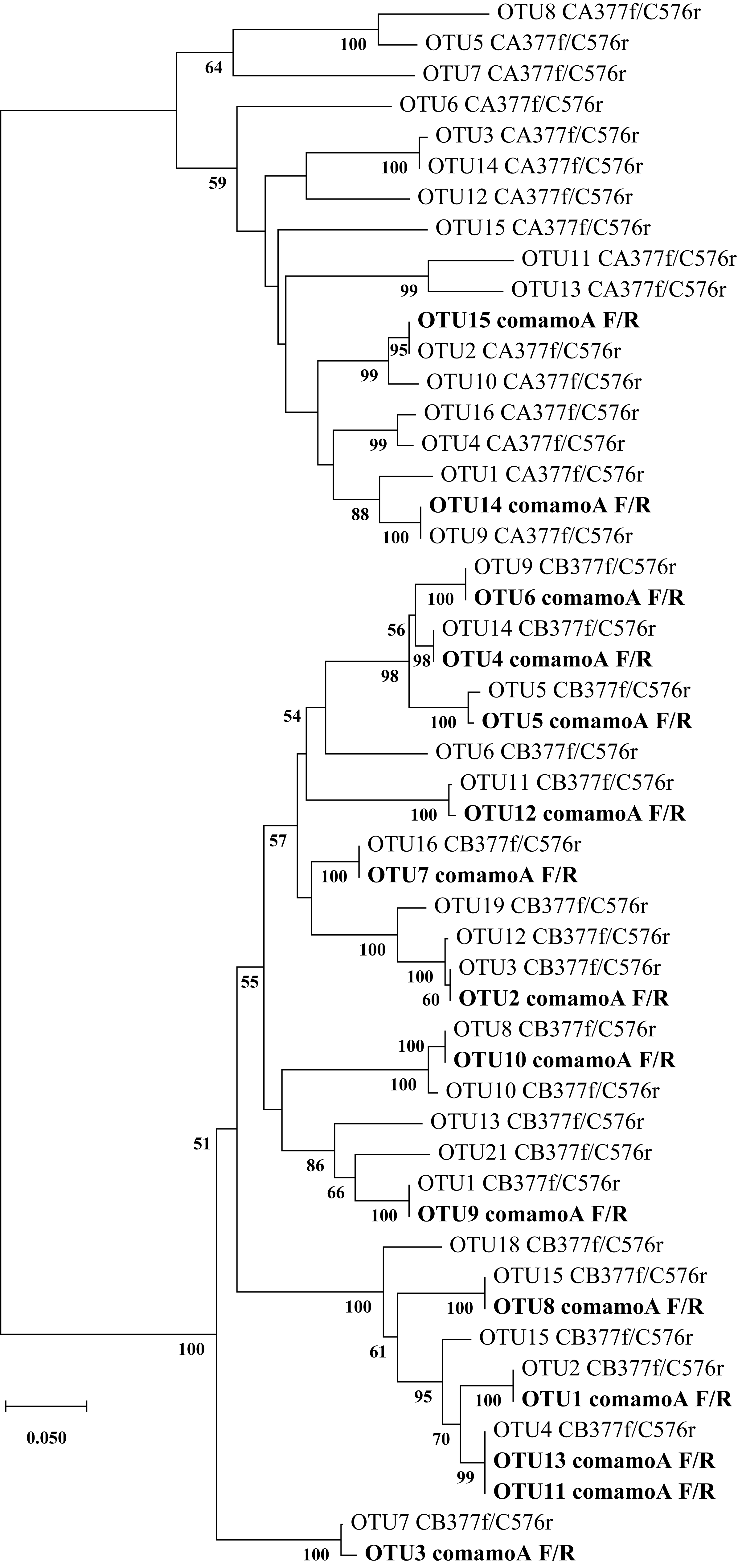


**Figure S4** Phylogenetic tree of comammox *amoA* gene sequences at the nucleotide level detected by primer sets comamoA F/R and CA/B377f/C576r. Only OTUs with more than 0.5% of the obtained comammox *amoA* gene sequences are shown in the phylogenetic tree. Phylogenetic analysis was performed using the neighbor-joining method with 1000 bootstraps. The scale bar represents 5% nucleic acid sequence divergence, and the bootstrap values (>50%) are shown at branch points.
