# Supplementary material for: Evaluation of four primer sets for analysis of comammox communities in black soils
Source: Front Microbiol. 2022 Jul 26;13:944373. doi: 10.3389/fmicb.2022.944373 (PMC9362984; doi:10.3389/fmicb.2022.944373)
Supplement: Supplementary file 2 [file Data_Sheet_2.docx]

Table S1 Properties of the comammox *amoA*-targeted primer sets used for PCR in this study.

| Primer Name | Primer sequence (5′-3′) | Target gene | Thermal profile | Amplification product length (bp) | Reference |
| --- | --- | --- | --- | --- | --- |
| Ntsp-amoA 162F  Ntsp-amoA 359R | GGATTTCTGGNTSGATTGGA  WAGTTNGACCACCASTACCA | Total comammox *amoA* | 95℃, 10min; 40 × (95℃, 45s; 48℃, 30s; 72℃, 45s); 72℃, 10min. | 198 | Fowler et al., 2018 |
|  |  |  |  |  |  |
| comamoA F  comamoA R | AGGNGAYTGGGAYTTCTGG  CGGACAWABRTGAABCCCAT | Total comammox *amoA* | 95℃, 15min; 40 × (95℃, 30s; 53℃, 30s; 72℃, 60s); 72℃, 10min. | 436 | Zhao et al., 2019 |
|  |  |  |  |  |  |
| CA377f  C576r | GTG GTG GTG GTC BAA YTA  GAA GCC CAT RTA RTC NGC C | Comammox Clade A *amoA* | 95℃, 10min; 40 × (94℃, 30s; 52℃, 45s; 72℃, 60s); 72℃, 10min. | 200 | Jiang et al., 2020 |
|  |  |  |  |  |  |
| CB377f  C576r | GTA CTG GTG GGC BAA YTT  GAA GCC CAT RTA RTC NGC C | Comammox Clade B *amoA* | 95℃, 10min; 40 × (94℃, 30s; 52℃, 45s; 72℃, 60s); 72℃, 10min. | 200 | Jiang et al., 2020 |

Degenerate bases: R, A/G; Y, C/T; W, A/T; S, G/C; B, G/C/T; and N, A/T/C/G.

Position is based on the *Nitrospira* sp. SG-bin1 *amoA* (GenBank, WP_011635097).

Table S2 The closest relative of dominant OTUs (relative abundance > 0.5%) with the primer set Ntsp-amoA 162F/659R at the amino acid level.

| OTU name | Length | Alignment | Closet relatives | Accession number | Identity (%) | Remarks |
| --- | --- | --- | --- | --- | --- | --- |
| OTU1 | 52 | 52/52 | Clone OTU_78 | QOQ37910 | 100 | Soil (Wang et al., 2020) |
| OTU2 | 52 | 52/52 | Clone N3P1K1_15 | QCX36502 | 100 | Paddy soil (Wang, unpublished) |
| OTU3 | 52 | 52/52 | Clone N3P1K1_15 | QCX36502 | 100 | Paddy soil (Wang and Wang, unpublished) |
| OTU4 | 52 | 52/52 | Clone N3P1K1_15 | QCX36502 | 100 | Paddy soil (Wang, unpublished) |
| OTU5 | 52 | 52/52 | Clone 162F/359ROTU222 | QJU69523 | 100 | Wetland soil (Wang, unpublished) |
| OTU6 | 52 | 52/52 | Clone OTU_40 | QOQ37872 | 100 | Soil (Wang et al., 2020) |
| OTU7 | 52 | 51/52 | Clone OTU_40 | QOQ37872 | 98 | Soil (Wang et al., 2020) |
| OTU8 | 52 | 52/52 | Clone 162F/364ROTU1009 | QJU69528 | 100 | Wetland soil (Wang, unpublished) |
| OTU9 | 52 | 52/52 | Clone 162F/387ROTU862 | QJU69551 | 100 | Wetland soil (Wang, unpublished) |
| OTU10 | 52 | 52/52 | Clone N3P1K1_15 | QCX36502 | 100 | Paddy soil (Wang, unpublished) |
| OTU11 | 52 | 52/52 | Clone OTU_78 | QOQ37910 | 100 | Soil (Wang et al., 2020) |
| OTU12 | 52 | 52/52 | Clone 162F/364ROTU1009 | QJU69528 | 100 | Wetland soil (Wang, unpublished) |
| OTU13 | 52 | 52/52 | Clone OTU_40 | QOQ37872 | 100 | Soil (Wang et al., 2020) |
| OTU14 | 52 | 52/52 | Clone OTU_49 | QOQ37881 | 100 | Soil (Wang et al., 2020) |
| OTU15 | 52 | 52/52 | Clone 162F/364ROTU1009 | QJU69528 | 100 | Wetland soil (Wang, unpublished) |
| OTU16 | 52 | 52/52 | Clone OTU78 | QAU21461 | 100 | Environmental sample (Zheng, unpublished) |
| OTU17 | 52 | 52/52 | Clone 162F/364ROTU1009 | QJU69528 | 100 | Wetland soil (Wang, unpublished) |
| OTU18 | 52 | 52/52 | Clone 162F/393ROTU580 | QJU69554 | 100 | Wetland soil (Wang, unpublished) |
| OTU19 | 52 | 52/52 | Clone 162F/387ROTU862 | QJU69551 | 100 | Wetland soil (Wang, unpublished) |
| OTU20 | 52 | 51/52 | Clone OTU_65 | QOQ37897 | 98 | Soil (Wang et al., 2020) |

Table S3 The closest relative of dominant OTUs (relative abundance > 0.5%) with the primer set comamoA F/R at the amino acid level.

| OTU name | Length | Alignment | Closet relatives | Accession number | Identity (%) | Remarks |
| --- | --- | --- | --- | --- | --- | --- |
| OTU1 | 132 | 131/132 | Clone Late_DF_60_51_G12-3 | ACM46939 | 99 | Soil (Levine et al., 2011) |
| OTU2 | 132 | 132/132 | Clone Late_DF_62_56_2_4_2_19 | ACM46921 | 100 | Soil (Levine et al., 2011) |
| OTU3 | 132 | 131/132 | Clone WH16090500179 | QBO71470 | 99 | Soil (Jiang et al., unpublished) |
| OTU4 | 132 | 132/132 | Clone Late_DF_60_51_G3-3 | ACM46978 | 100 | Soil (Levine et al., 2011) |
| OTU5 | 132 | 128/132 | Clone Late_DF_62_56_1_2_D04 | ACM46914 | 97 | Soil (Levine et al., 2011) |
| OTU6 | 132 | 132/132 | Clone QTIM18-10-40 | AFI78851 | 100 | Alpine meadow and grassland soil (Rui et al., unpublished) |
| OTU7 | 132 | 131/132 | Clone XxxM0102 | AUT32062 | 99 | Upland soil (Deng et al., 2013) |
| OTU8 | 132 | 132/132 | Clone Late_DF_60_51_G12-3 | ACM46939 | 100 | Soil (Levine et al., 2011) |
| OTU9 | 132 | 130/132 | *Nitrospirae* bacterium isolate | MBI5317252 | 98 | Groundwater (He et al., 2020) |
| OTU10 | 132 | 132/132 | Clone Late_DF_62_56_1_4_D06 | ACM46915 | 100 | Soil (Levine et al., 2011) |
| OTU11 | 132 | 132/132 | Clone Late_DF_60_51_G12-3 | ACM46939 | 100 | Soil (Levine et al., 2011) |
| OTU12 | 132 | 131/132 | Clone CL33 | CAG27857 | 99 | Upland soil (Ricke et al., unpublished) |
| OTU13 | 132 | 131/132 | Clone Late_DF_60_51_G12-3 | ACM46939 | 99 | Soil (Levine et al., 2011) |
| OTU14 | 132 | 132/132 | Clone coma_amoA_A-1 | AYN63916 | 100 | Freshwater river sediment (Xia et al., 2018) |
| OTU15 | 132 | 132/132 | Clone OTU72 | QAU21455 | 100 | Environmental sample (Zheng, unpublished) |

Table S4 The closest relative of dominant OTUs (relative abundance > 0.5%) with the primer set CA377f/C576r at the amino acid level.

| OTU name | Length | Alignment | Closet relatives | Accession number | Identity (%) | Remarks |
| --- | --- | --- | --- | --- | --- | --- |
| OTU1 | 65 | 65/65 | Clone coma_amoA_169 | ASO97140 | 100 | Rhizosphere (rice) (Pjevac et al., 2017) |
| OTU2 | 65 | 65/65 | Clone coma_amoA_196 | ASO97167 | 100 | Paddy soil (Pjevac et al., 2017) |
| OTU3 | 65 | 64/65 | Clone coma_amoA_169 | ASO97140 | 98 | Rhizosphere (rice) (Pjevac et al., 2017) |
| OTU4 | 65 | 65/65 | Clone coma_amoA_169 | ASO97140 | 100 | Rhizosphere (rice) (Pjevac et al., 2017) |
| OTU5 | 65 | 65/65 | Clone coma_amoA_80 | ASO97051 | 100 | Drinking water treatmant plant (Pjevac et al., 2017) |
| OTU6 | 65 | 65/65 | Clone coma_amoA_182 | ASO97153 | 100 | Rhizosphere (rice) (Pjevac et al., 2017) |
| OTU7 | 65 | 65/65 | Clone coma_amoA_202 | ASO97173 | 100 | Paddy soil (Pjevac et al., 2017) |
| OTU8 | 65 | 65/65 | Clone coma_amoA_3 | ASO96975 | 100 | Groundwater well (Pjevac et al., 2017) |
| OTU9 | 65 | 65/65 | Clone coma_amoA_169 | ASO97140 | 100 | Rhizosphere (rice) (Pjevac et al., 2017) |
| OTU10 | 65 | 65/65 | Clone coma_amoA_196 | ASO97167 | 100 | Paddy soil (Pjevac et al., 2017) |
| OTU11 | 65 | 65/65 | Clone coma_amoA_171 | ASO97142 | 100 | Rhizosphere (rice) (Pjevac et al., 2017) |
| OTU12 | 65 | 65/65 | Clone com-cladeA-OTU30 | QXU65144 | 100 | Estuaries tidal flat wetland (Sun et al., 2021) |
| OTU13 | 65 | 65/65 | Clone coma_amoA_171 | ASO97142 | 100 | Rhizosphere (rice) (Pjevac et al., 2017) |
| OTU14 | 65 | 64/65 | Clone coma_amoA_169 | ASO97140 | 98 | Rhizosphere (rice) (Pjevac et al., 2017) |
| OTU15 | 65 | 65/65 | Clone FD12 | QPD98996 | 100 | Forest soil (Li et al., unpublished) |
| OTU16 | 65 | 65/65 | Clone coma_amoA_169 | ASO97140 | 100 | Rhizosphere (rice) (Pjevac et al., 2017) |

Table S5 The closest relative of dominant OTUs (relative abundance > 0.5%) with the primer set CB377f/C576r at the amino acid level.

| OTU name | Length | Alignment | Closet relatives | Accession number | Identity (%) | Remarks |
| --- | --- | --- | --- | --- | --- | --- |
| OTU1 | 65 | 65/65 | Clone coma_amoA_410 | ASO97378 | 100 | Recirculating aquarium filter (Pjevac et al., 2017) |
| OTU2 | 65 | 65/65 | Clone coma_amoA_213 | QJU69560 | 100 | Wetland soil (Pjevac et al., 2017) |
| OTU3 | 65 | 65/65 | Clone coma_amoA_241 | ASO97212 | 100 | Rhizosphere (rice) (Pjevac et al., 2017) |
| OTU4 | 65 | 65/65 | Clone coma_amoA_213 | QJU69560 | 100 | Wetland soil (Wang., unpublished) |
| OTU5 | 65 | 65/65 | Clone coma_amoA_229 | ASO97200 | 100 | Forest soil (Pjevac et al., 2017) |
| OTU6 | 65 | 65/65 | Clone comaA/B-244f/669rOTU102 | QJU69567 | 100 | Wetland soil (Wang., unpublished) |
| OTU7 | 65 | 65/65 | Clone coma_amoA_265 | ASO97236 | 100 | Rhizosphere (rice) (Pjevac et al., 2017) |
| OTU8 | 65 | 65/65 | Clone coma_amoA_335 | ASO97306 | 100 | Forest soil (Pjevac et al., 2017) |
| OTU9 | 65 | 65/65 | Clone QTIM18-10-40 | AFI78851 | 100 | Alpine meadow and grassland soil (Rui., unpublished) |
| OTU10 | 65 | 65/65 | Clone coma_amoA_335 | ASO97306 | 100 | Forest soil (Pjevac et al., 2017) |
| OTU11 | 65 | 65/65 | Clone CL1_04 | CAH10440 | 100 | Upland soils (Kolb et al., 2005) |
| OTU12 | 65 | 65/65 | Clone coma_amoA_241 | ASO97212 | 100 | Rhizosphere (rice) (Pjevac et al., 2017) |
| OTU13 | 65 | 65/65 | Clone com-cladeB-OTU82 | QXU65271 | 100 | Estuaries tidal flat wetland (Sun et al., 2021) |
| OTU14 | 65 | 65/65 | Clone coma_amoA_213 | ASO97184 | 100 | Forest soil (Pjevac et al., 2017) |
| OTU15 | 65 | 65/65 | Clone coma_amoA_213 | QJU69560 | 100 | Wetland soil (Pjevac et al., 2017) |
| OTU16 | 65 | 65/65 | Clone UT-top-AA2 | AEZ02343 | 100 | Upland tundra top soil (Martineau et al., 2014) |
| OTU17 | 65 | 64/65 | Clone coma_amoA_263 | ASO97234 | 98 | Rhizosphere (rice) (Pjevac et al., 2017) |
| OTU18 | 65 | 65/65 | Clone com-cladeB-OTU94 | QOD39744 | 100 | Estuaries tidal flat wetland (Sun., unpublished) |
| OTU19 | 65 | 65/65 | Clone coma_amoA_257 | ASO97228 | 100 | Rhizosphere (rice) (Pjevac et al., 2017) |
| OTU20 | 65 | 65/65 | Clone coma_amoA_213 | QJU69560 | 100 | Wetland soil (Wang., unpublished) |
| OTU21 | 65 | 65/65 | Clone coma_amoA_410 | ASO97378 | 100 | Recirculating aquarium filter (Pjevac et al., 2017) |

Table S6 Sequence numbers and percentages of different *amoA* gene sequences.

| Sample  name | Ntsp-amoA 162F/359R | | |  | comamoA F/R | | | |  | CA377f/C576r | | |  | CB377f/C576r | |
| --- | --- | --- | --- | --- | --- | --- | --- | --- | --- | --- | --- | --- | --- | --- | --- |
|  | Total | Clade A | Clade B |  | Total | Clade A | Clade B | AOB |  | Total | Clade A | AOB |  | Total | Clade B |
| N1-1 | 12645 | 79 (1%) | 12566 (99%) |  | 3603 | 25 (1%) | 3576 (99%) | 2 (0%) |  | 4185 | 4185 (100%) | / |  | 2434 | 2434 (100%) |
| N1-2 | 12645 | 479 (4%) | 12166 (96%) |  | 3603 | 91 (3%) | 3511 (97%) | 1 (0%) |  | 4185 | 4183 (100%) | 2(0%) |  | 2434 | 2434 (100%) |
| N1-3 | 12645 | 1335 (11%) | 11310 (89%) |  | 3603 | 56 (2%) | 3545 (98%) | 2 (0%) |  | 4185 | 4183 (100%) | 2(0%) |  | 2434 | 2434 (100%) |
| F1-1 | 12645 | 932 (7%) | 11713 (93%) |  | 3603 | 233 (6%) | 3369 (94%) | 1 (0%) |  | 4185 | 4185 (100%) | / |  | 2434 | 2434 (100%) |
| F1-2 | 12645 | 100 (1%) | 12545 (99%) |  | 3603 | 436 (12%) | 3158 (88%) | 9 (0%) |  | 4185 | 4185 (100%) | / |  | 2434 | 2434 (100%) |
| F1-3 | 12645 | 728 (6%) | 11917 (94%) |  | 3603 | 113 (3%) | 3490 (97%) | / |  | 4185 | 4185 (100%) | / |  | 2434 | 2434 (100%) |
| N2-1 | 12645 | 301 (2%) | 12344 (98%) |  | 3603 | 15 (0%) | 3588 (100%) | / |  | 4185 | 4185 (100%) | / |  | 2434 | 2434 (100%) |
| N2-2 | 12645 | 451 (4%) | 12194 (96%) |  | 3603 | 140 (4%) | 3396 (94%) | 67 (2%) |  | 4185 | 4185 (100%) | / |  | 2434 | 2434 (100%) |
| N2-3 | 12645 | 409 (3%) | 12236 (97%) |  | 3603 | 43 (1%) | 3549 (99%) | 11 (0%) |  | 4185 | 4185 (100%) | / |  | 2434 | 2434 (100%) |
| F2-1 | 12645 | 214 (2%) | 12431 (98%) |  | 3603 | 50 (1%) | 3553 (99%) | / |  | 4185 | 4185 (100%) | / |  | 2434 | 2434 (100%) |
| F2-2 | 12645 | 1 (0%) | 12644 (100%) |  | 3603 | 249 (7%) | 3287 (91%) | 67 (2%) |  | 4185 | 4177 (100%) | 8(0%) |  | 2434 | 2434 (100%) |
| F2-3 | 12645 | 132 (1%) | 12513 (99%) |  | 3603 | 102 (3%) | 3490 (97%) | 11 (0%) |  | 4185 | 4184 (100%) | 1(0%) |  | 2434 | 2434 (100%) |

N1 and F1 are natural soils and arable soils in Hongwuyue farm, respectively; N2 and F2 are natural soils and arable soils in Zhaoguang farm, respectively. Each treatment has three replicates.

**Supplementary references**

Deng, Y., Cui, X., Luke, C., Dumont, M.G., (2013). Aerobic methanotroph diversity in Riganqiao peatlands on the Qinghai-Tibetan Plateau. *Environ. Microbiol. Rep.* 5, 566-574. doi: 10.1111/1758-2229.12046

Fowler, S.J., Palomo, A., Dechesne, A., Mines, P.D., Smets, B.F., (2018). Comammox *Nitrospira* are abundant ammonia oxidizers in diverse groundwater-fed rapid sand filter communities. *Environ. Microbiol.* 20, 1002-1015. doi: 10.1111/1462-2920.14033

He, C., Keren, R., Keren, R., Whittaker, M., Farag, I.F., Doudna, J., Cate, J.H.D., Banfield, J., (2021). Genome-resolved metagenomics reveals site-specific diversity of episymbiotic CPR bacteria and DPANN archaea in groundwater ecosystems. *Nat. Microbiol.* 6, 354-365. doi: 10.1038/s41564-020-00840-5

Jiang, R., Wang, J.G., Zhu, T., Zou, B., Wang, D.Q., Rhee, S.K., An, D., Ji, Z.Y., Quan, Z.X., (2020). Use of Newly Designed Primers for Quantification of Complete Ammonia-Oxidizing (Comammox) Bacterial Clades and Strict Nitrite Oxidizers in the Genus Nitrospira. *Appl. Environ. Microbiol.* 86, e01775-20. doi: 10.1128/AEM.01775-20

Kolb, S., Knief, C., Dunfield, P.F., Conrad, R., (2005). Abundance and activity of uncultured methanotrophic bacteria involved in the consumption of atmospheric methane in two forest soils. *Environ. Microbiol.* 7, 1150-1161. doi: 10.1111/j.1462-2920.2005.00791.x

Levine, U.Y., Teal, T.K., Robertson, G.P., Schmidt, T.M., (2011). Agriculture's impact on microbial diversity and associated fluxes of carbon dioxide and methane. *ISME J.* 5, 1683-1691. doi: 10.1038/ismej.2011.40

Pjevac, P., Schauberger, C., Poghosyan, L., Herbold, C.W., van Kessel, M., Daebeler, A., Steinberger, M., Jetten, M.S.M., Lucker, S., Wagner, M., Daims, H., 2017. *AmoA*-Targeted Polymerase Chain Reaction Primers for the Specific Detection and Quantification of Comammox *Nitrospira* in the Environment. Front Microbiol. 8, 1508. doi: 10.3389/fmicb.2017.01508

Sun, D., Zhao, M., Tang, X., Liu, M., Hou, L., Zhao, Q., Li, J., Gu, J.D., Han, P., (2021). Niche adaptation strategies of different clades of comammox *Nitrospira* in the Yangtze Estuary. *Int. Biodeter. Biodegr.* 164, 105286. doi: 10.1016/j.ibiod.2021.105286

Xia, F., Wang, J.G., Zhu, T., Zou, B., Rhee, S.K., Quan, Z.X., (2018). Ubiquity and Diversity of Complete Ammonia Oxidizers (Comammox). *Appl. Environ. Microbiol.* 84, e01390-18. doi: 10.1128/AEM.01390-18

Wang, X., Lu, L., Zhou, X., Tang, X., Kuang, L., Chen, J., Shan, J., Lu, H., Qin, H., Adams, J., Wang, B., (2020). Niche Differentiation of Comammox *Nitrospira* in the Mudflat and Reclaimed Agricultural Soils Along the North Branch of Yangtze River Estuary. *Front. Microbiol.* 11, 618287. [doi: 10.3389/fmicb.2020.618287](https://doi.org/10.3389/fmicb.2020.618287)

Zhao, Z., Huang, G., He, S., Zhou, N., Wang, M., Dang, C., Wang, J., Zheng, M., (2019). Abundance and community composition of comammox bacteria in different ecosystems by a universal primer set. *Sci. Total Environ.* 691, 146-155 doi: 10.1016/j.scitotenv.2019.07.131
